# Supplementary material for: Myofascial trigger point (MTrP) size and elasticity properties can be used to differentiate characteristics of MTrPs in lower back skeletal muscle
Source: Sci Rep. 2024 Mar 30;14:7562. doi: 10.1038/s41598-024-57733-4 (PMC10981696; doi:10.1038/s41598-024-57733-4)
Supplement: Supplementary file 1 — Supplementary Tables. [file 41598_2024_57733_MOESM1_ESM.pdf]

Supplementary Tables:

Stiffness	1	2	3	4
1	-	0.2	0.94	0.45
2		-	0.31	0.46
3			-	0.57
4				-

Table S1: Statistical P-values for Stiffness by group

Thickness	1	2	3	4
1	-	0.57	0.98	0.37
2		-	0.6	0.2
3			-	0.39
4				-

Table S2: Statistical P-values for Thickness by group

Depth	1	2	3	4
1	-	0.72	0.01	0.06
2		-	0.07	0.27
3			-	0.19
4				-

Table S3: Statistical P-values for Depth by group

Strain	1	2	3	4
1	-	0.14	0.61	0.27
2		-	0.42	0.48
3			-	0.69
4				-

Table S4: Statistical P-values for depth corrected strain by group
